# Regulation of Ca^2+^ transient by PP2A in normal and failing heart

**DOI:** 10.3389/fphys.2015.00013

**Published:** 2015-01-29

**Authors:** Ming Lei, Xin Wang, Yunbo Ke, R. John Solaro

**Affiliations:** ^1^Department of Pharmacology, University of OxfordOxford, UK; ^2^Faculty of Life Science, University of ManchesterManchester, UK; ^3^Department of Physiology and Biophysics, Center for Cardiovascular Research, University of Illinois at ChicagoChicago, IL, USA

**Keywords:** calcium handling, ion channels, phosphatase, FTY720, arrhythmia, heart failure

## Abstract

Calcium transient in cardiomyocytes is regulated by multiple protein kinases and phosphatases. PP2A is a major protein phosphatase in the heart modulating Ca^2+^ handling through an array of ion channels, antiporters and pumps, etc. The assembly, localization/translocation, and substrate specificity of PP2A are controlled by different post-translational mechanisms, which in turn are linked to the activities of upstream signaling molecules. Abnormal PP2A expression and activities are associated with defective response to β-adrenergic stimulation and are indication and causal factors in arrhythmia and heart failure.

## Introduction

Cyclic and effective cardiac contraction and relaxation depend on the appropriately timed generation and spread of cardiac electrical activity. At the cellular level, excitation-contraction (E-C) coupling is initiated by action potential depolarization resulting, via a cascade of events, in an increase in intracellular calcium concentration, which ultimately leads to activation of myofilament and muscle contraction; subsequent removal of intracellular calcium via a number of mechanisms results in detachment of myosin cross-bridges and relaxation. Excitation and contraction involve multiple trans-membrane (e.g., ion channels) and intracellular proteins (e.g., Ca^2+^ handling and sarcomeric proteins) and are highly regulated by multiple extra- and intra-cellular signaling pathways that frequently converge at protein phosphorylation.

Studies of reversible protein phosphorylation in the heart date back to early seventies of last century when it was reported that cardiac troponin I (cTnI) was phosphorylated and dephosphorylated in the same manner as the protein substrates involved in glycogen metabolism (England et al., 1972; Stull et al., 1972). cTnI is the inhibitory component of heterotrimeric troponin complex and a major phosphoprotein in ventricular myocytes. cAMP dependent protein kinase (PKA), a downstream effector of β-adrenergic stimulations, phosphorylates cTnI at serine 23 and 24 (Cole and Perry, 1975; Solaro et al., 1976). Phosphorylation of cTnI promotes Ca^2+^ release from the myofilament and promotes cardiac relaxation (Robertson et al., 1982; Kentish et al., 2001). PP2A came into spotlight of heart research following another line of observation in late 1980s and early 1990s. It was found that an extract from black sea sponge, okadaic acid, has positive inotropic effect on electro-mechanic properties of ventricular muscle and enhances pacemaker activities in rabbit SA node preparation (Kodama et al., 1986; Kondo et al., 1990). Okadaic acid inhibits protein phosphatase PP2A at very low concentration leading to increased phosphorylation in numerous proteins of mammalian cells, including a number of ion channels and myofilament regulatory proteins. Thus, PP2A coordinates cardiac excitation and contraction.

The catalytic subunit of PP2A is highly conserved from yeast to humans and is homologous to the counterpart of PP1 complex, another major protein phosphatase in mammalian cells, which consists of catalytic and regulatory/targeting subunit with more than more than 200 isoforms (Depaoli-Roach et al., 1994; Peti et al., 2013). PP1 and PP2A are responsible for greater than 90% of protein dephosphorylation in the heart and they often share the same protein substrates and serine/threonine sites of dephosphorylation (Luss et al., 2000). However, their relative contributions to specific protein substrates o are often different, which is reflected in dephosphoryation of L-type Ca^2+^ channels (PP2A preferred) and phospholamban (PP1 preferred). For a long time, mammalian protein phosphatases had been considered constitutively active with the regulatory function fulfilled solely by protein kinases. This notion has become obsolete with discovery of multiple regulatory mechanisms for protein phosphatases, especially those that link phosphatase activities to extracellular cues (Cohen, 1988). The importance of regulation of phosphatases in heart pathophysiology becomes more obvious when altered PP2A expression and activities are closely associated with heart diseases (Ai and Pogwizd, 2005; Ke et al., 2008; Wijnker et al., 2011).

## PP2A and its regulation by upstream signals in the heart

**Figure 1:**
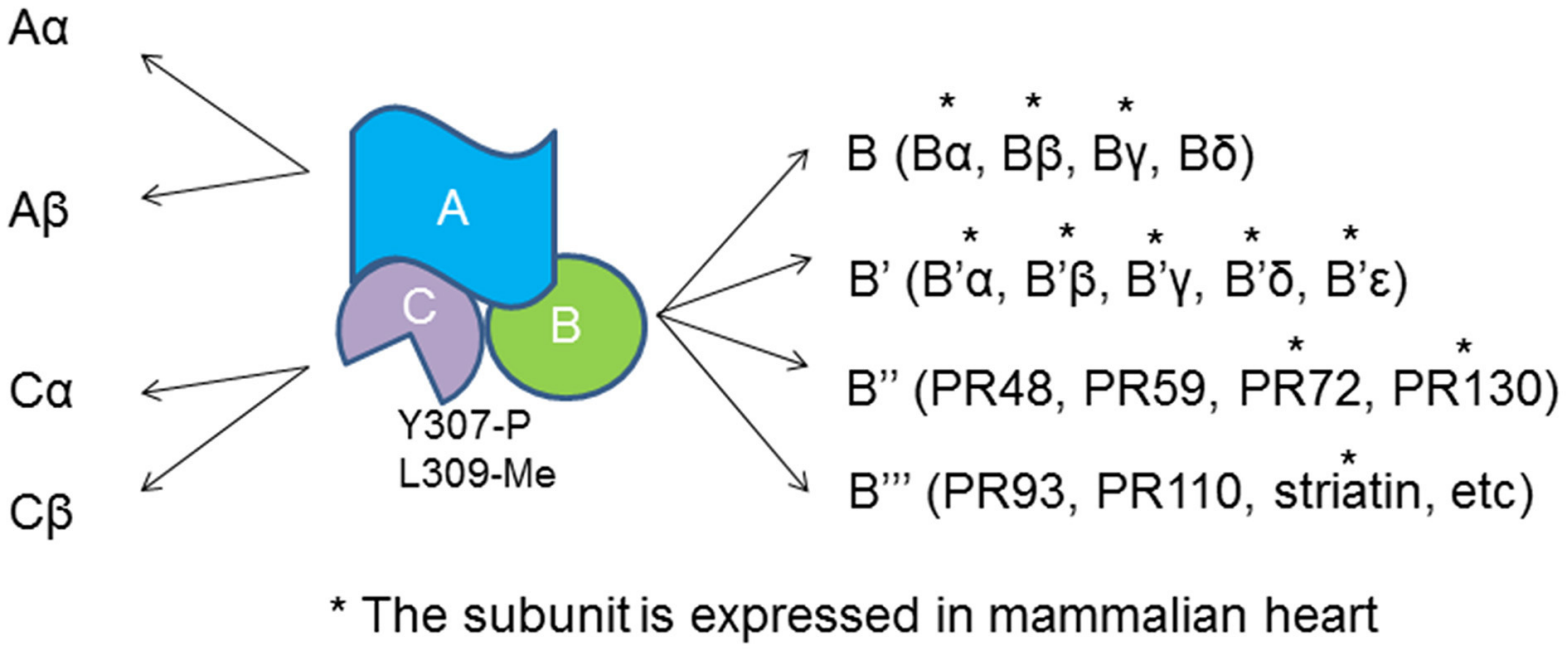
**PP2A heterotrimer and the subunits**. Both A and C subunit have two isoforms, α and β. The catalytic C subunit can be tyrosine phosphorylated at tyrosine 307 and methylated at Leucine 309. ^*^The B subunit is expressed and identified in cardiomyocytes.

PP2A actually refer to a large family of distinct heterotrimeric protein phosphatases that share a common core enzyme consisting of a scaffolding (A) and a catalytic (C) subunits that associate with a B subunit (Figure 1). A subunit contains multiple HEAT repeats and forms a horse shoe structure that bind to both B and C subunits (Groves et al., 1999). HEAT repeat exists in proteins with different functions that form helical structures and provide structural flexibility to PP2A-A subunit (Grinthal et al., 2010). Formation of the PP2A heterotrimer follows a sequential pattern in that the core enzyme AC arises first and then binds to the B subunit. The Tyrosine 307 and Leucine 309 show reversible phosphorylation and methylation that determine the phosphatase localization and substrate specificity (Chen et al., 1992; Chung et al., 1999). Methylation of Leucine 309 diverts the C-terminal carboxyl group from a repulsive negative charge interaction and facilitates assembly of ABC holoenzyme (Cho and Xu, 2007).

The regulatory subunits of PP2A have many members with large sequence diversity and are coded by at least 17 distinct genes. At least 11 of them are expressed in cardiomyocytes with Bα and γ the most studied cardiac isoforms (Figure 1). Bα is abundant in cytoplasm in cardiomyocyte that associates with ankyrin-B, an adapter protein required for normal subcellular localization of the Na/Ca exchanger, Na/K ATPase (Bhasin et al., 2007). Overexpression of Bα leads to reduced phosphorylation cTnI, myosin-binding protein C and phospholamban, and repressed response of L-type Ca^2+^ channel current to stimulation of isoproterenol (Kirchhefer et al., 2014a). Bγ is expressed in the nuclear. In mouse model deficient in Bγ, an incomplete ventricular septum occurs during development. PR72 binds to Ca^2+^ resulting in conformational changes in the scaffolding subunit. Another Ca^2+^ responsive B subunit expressed in cariomyocytes is striatin that directly interacts with calmodulin (Chen et al., 2014; Hwang and Pallas, 2014). It remains unclear if PP2As containing these B subunits control cyclic dephophorylation on any protein substrates. A genome wide association studies has identified a deletion mutation that links abnormal striatin mRNA accumulation to arrhythmogenic right ventricular cardiomyopathy in canine model (Meurs et al., 2010).

Both PP1 and PP2A have native inhibitors in mammalian cells. Inhibitor I of PP1 is a phosphoprotein regulated by β-adrenergic stimulation and is important for modulation of Ca^2+^ re-uptake through phospholamban. I1 and I2 PP2A are specific PP2A inhibitors (Li et al., 1995). Their expression and functional role in cardiomyocytes is underexplored. PP2A is also up-regulated by small molecular weight chemicals, both native and artificial. C_2_ and C_6_ ceramides activates PP2A in different types of mammalian cells (Dobrowsky et al., 1993). FTY720 (fingolimod) is a synthetic analog of C_2_ and C_6_ ceramide and an immunosuppressor used for treatment of multiple schlerosis (Kappos et al., 2006). Like C_2_ and C_6_ ceramide, FTY720 activates PP2A without knowing exactly what the molecular mechanism of activation. P^21^ activated kinase-1 (Pak1), an upstream activator for PP2A, is activated by FTY720 and C2/C6 ceramides *on vitro* and *in vivo* (Ke and Solaro, 2008; Egom et al., 2010; Liu et al., 2011b).

**Figure 2:**
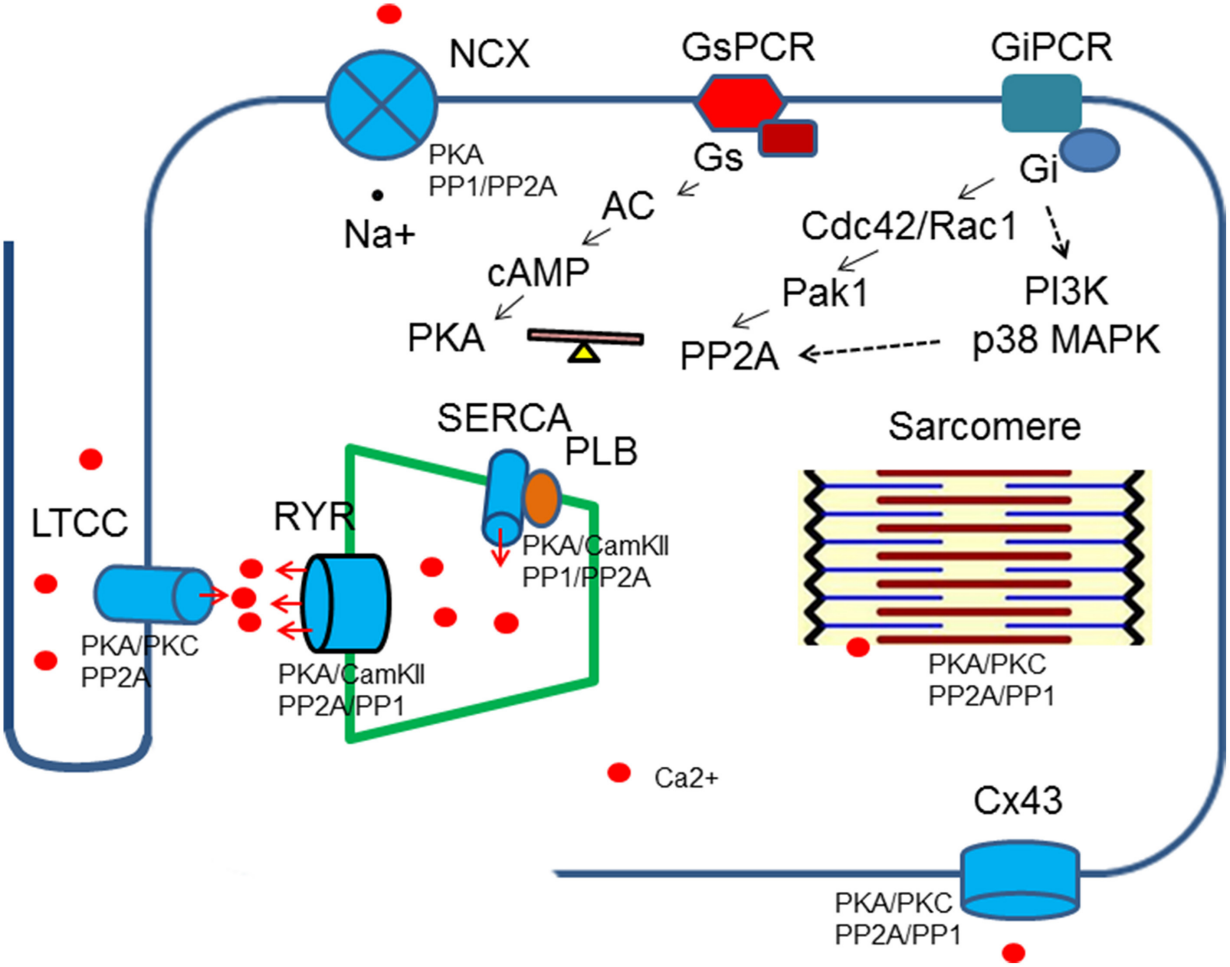
**Regulation of Ca^2+^ transient by protein kinases and phosphatases**. Protein kinases and phospahtases are associated with key Ca^2+^ transient regulatory proteins, which in turn are linked to upstream signaling cascades. A balance of protein kinase and phosphatase activities is required to maintain normal cardiac functions. Breakdown of the balance occurs at different levels: genetic mutations, gene expressions, post-translational modifications and excessive or deficient neuro-hormonal cues.

Accumulating evidence has indicated that PP2A activities are up-regulated by stimulation of the inhibitory G proteins, Gi through different intermediate signaling processes (Ke et al., 2008). Treatment of ventricle cardiomyocytes with agonists that turn on receptors coupled to inhibitory G proteins (Gi/Go) leads to reduced phosphorylation on PKA substrates without any change in intracellular cAMP, suggesting phosphatases are responsible for reduction in protein phosphorylation (Gupta et al., 1993, 1994). In cardiomyocytes, methylation of PP2Ac is reduced when the cells are treated with pertussis toxin and the same result is generated by inhibition of p38 MAP kinase (Liu and Hofmann, 2002, 2003). Cdc42 and Rac1 have been shown to be the downstream effectors for Gi in cardiomycytes and other mammalian cells. The constitutively active Pak1, the downstream effectors for Cdc42 and Rac1 induces activation of PP2A and dephosphorylation of myofilament regulatory proteins (Ke et al., 2004). PI3K is another possible link between Gi and PP2A activities that enhances carboxylmethylation at leu309 (Longman et al., 2014) (Figure 2).

## Regulation of Ca^2+^ handling proteins by PP2A

The calcium transient starts through depolarization-activated Ca^2+^ channels. The inward calcium current triggers Ca^2+^ release from the sarcoplasmic reticulum mediated primarily by ryanodine receptors. The Ca^2+^ binds to troponin C of troponin/tropomyosin complex and activates myofilaments. During relaxation, cytosolic Ca^2+^ is pumped back into sarcoplasmic reticulum by SR Ca ATPase (SERCA) and is removed from the cells by Na^+^/Ca^2+^ exchanger. Protein kinases and PP2A associate with all of these key regulatory machinery and shape the dynamics of Ca^2+^ flow (Table 1, Figure 2).

**Table 1:** **Major targets regulating Ca^2+^ transient and regulated by PP2A**.

**Targets**	**Reported phosphorylation sites**	**Protein kinases**	**Protein phosphatases**	**Effects of PP2A on channel activities**	**References**
L type Ca^2+^ channels	Ser1928	PKA	PP2A	↓	Chen et al., 2002; Hall et al., 2006
	Ser1866				Davare et al., 2000; Shi et al., 2012
Ryanodine receptors	Ser2808	PKA, CamKII	PP2A	↓↑	Marx et al., 2000; Xiao et al., 2005, 2006; Meng et al., 2007; Liu et al., 2011a; Zhang et al., 2012
	Ser2030		PP1		Liu et al., 2014
Phospho- lamban	Ser16 and Thr17	PKA CamKII	PP1 PP2A	Release of inhibition on SERCA	MacDougall et al., 1991; Luo et al., 1994; Jackson and Colyer, 1996; Chu and Kranias, 2002
Connexin 43	Ser368 Ser262	PKC PKA	PP2A PP1	↓	Doble et al., 2000; Ai and Pogwizd, 2005; Srisakuldee et al., 2009
NCX	?	PKA	PP2A	↓?	Wei et al., 2003, 2007
		PKC	PP1		Schulze et al., 2003; Zhang and Hancox, 2009

### PP2A is a major phosphatase for L-type Ca^2+^ channels (LTCC)

The voltage gated influx of Ca^2+^ through LTCC is highly responsive to β-adrenergic stimulation. PKA phosphorylates LTCC at the cytoplasmic, carboxyl end of alpha subunit of LTCC at Ser1928, Ser1866 (Chen et al., 2002; Hall et al., 2006), phosphorylation of S1512 and S1570 by Cam Kinase II may also play an auxiliary role modulating the channel activities (Blaich et al., 2010). The β-adrenergic effect on LTCC is reversed by PP2A, which associates with the channels at the PKA sites (Davare et al., 2000). In pacemaker cells, activation of PP2A by its upstream signal, Pak1, represses isoproterenol stimulated enhancement of the channel activities (Ke et al., 2007).

### The roles of PP2A on ryanodine receptor (RyR) regulation

Ca^2+^ induced Ca^2+^ release through LTCC and ryanodine receptors is enhanced by β-adrenergic signaling cascades. Ser2808 and Ser2030 are considered as the PKA sites. Early studies suggest that hyperphosphorylation of RyR at Ser 2808 is responsible for increased leak for Ca^2+^ and associated with heart failure. Surprisinglya recent study has shown that in genetically modified mice with Ser2808 rendered unphosphorylatable, Ca^2+^ leak increases, instead of decrease with exacerbation of Ca^2+^-dependent cardiomyopathy (Liu et al., 2014). On the other hand, Yang et al. recently indicate that a reduced degradation of β2-AR due to Rnd3 deficiency results in enhanced PKA activities and increased Ca^2+^ leak from RyR (Yang et al., 2015). PP1 and PP2A form complexes on ryanodine receptors. In saponin permeabilized myocytes, exposure of PP1 and PP2A dramatically increased Ca sparks with a significant decrease of SR Ca store (Terentyev et al., 2003). On the other hand, targeting of PP2A regulatory subunit B56α by microRNA miR-1 leads to hyperphosphorylation of RyR at the CamKII sites and increases Ca^2+^ release and promote cardiac arrhythmogenesis (Terentyev et al., 2009; Belevych et al., 2011). PP2A is also responsible for dephosphorylation of RyR from the CamKII sites which have now been considered to play an even more important roles enhancing Ca^2+^ leak from the channel.

### PP2A is not a major protein phosphatase for phospholamban

SERCA, a calcium transport ATPase for Ca^2+^ reuptake from cytosol to SR partners with phospholamban that is phosphorylated at Ser16 and threonine17 by PKA and CamKII, respectively. Phospholamban inhibits SERCA activities and the inhibition is released by PKA phosphorylation and Ser16. PP1 is the major phosphatase that removes phosphate from both locations. PP2A plays a minor role (30%) of dephosphorylation (MacDougall et al., 1991). In mice with overexpression of the regulatory subunit of PP2A, the isoproterenol stimulated phosphorylation of phosholamban and cTnI is partially reduced with increased basal contractility of the heart, likely due to elevated diastolic Ca^2+^ level and increased myofilament activities (Kirchhefer et al., 2014a).

### The activities of connexin 43 are inhibited by PP2A

The gap junction channel protein connexin 43 conducts ions and other small molecules between two adjacent myocytes. The conductivity of connexin 43 is enhanced by PKA and reduced by PP2A as demonstrated by intercellular dye coupling (Ai and Pogwizd, 2005; Ai et al., 2011).

### PP2A and Na/Ca exchanger

The cardiac Na/Ca^2+^ exchanger (NCX) is involved in the extrusion of cytosolic Ca^2+^ with a major role in the decay phase of the intracellular Ca^2+^ transient. PP1 and PP2A form complex with Na/Ca exchanger (Schulze et al., 2003). Stimulation of PKA activities by dibutyryl cyclic AMP and inhibition of PP2A by okadaic acid inhibits NCX activities (Lin et al., 1994). However, studies from other groups reported mixed results regarding the role of β-adrenergic stimulation on NCX activities (Zhang and Hancox, 2009). Wei et al. indicated that hyperphosphorylation of NCX is associated with an increased NCX current. In failing heart, low phosphatase activity and hyperphosphorylation is responsible for impaired sensitivity to β-adrenergic stimulation (Wei et al., 2007).

## Aberrant expression, localization, and activities of PP2A in arrhythmia and heart failure

The importance of PP2A in the heart resides in its capacity to antagonize the effects of β-adrenergic stimulation with reduction of the amplitude of Ca transient and meanwhile increasing the Ca^2+^ sensitivity of myofilament in force development. Therefore, abnormality in PP2A expression, localization and activities are frequently associated with heart failure. However, the role of PP2A as a causal or beneficial factor in heart failure remains unclear.

### Expression and activities of PP2A in heart failure

In a rat model with chronic isoproterenol infusion that lead to cardiac hypertrophy and heart failure, PP2A activities increased significantly at day 2 (Boknik et al., 2000). In HF induced by tachypacing in sheep, increased PP1 and PP2A activities are associated with diminished response to β-adrenergic stimulation in amplitude of Ca^2+^ transient compared to normal heart (Briston et al., 2011). Overexpression of the catalytic subunit of PP2A (PP2A-C) by transgenic approach in mouse heart leads to left ventricular hypertrophy and reduced contractility along with an increase of PP2A activities in myocardium (Gergs et al., 2004). A more detailed analysis of expression and localization of different PP2A B subunits in cardiomyocytes from normal and failing hearts indicate that proper targeting and localization of PP2A holoenzyme are important for normal cardiac functions (DeGrande et al., 2013). On the other hand, in human heart with ischemic cardiomyopathy (ICM) and dilated cardiomyopathy (DCM), expression of both PP2A-C and PP2A-B α are reduced by half or more compared to the non-failing heart. Studies in transgenic mice over-expressing the regulatory subunit Bα indicate that this subunit directs PP2A core enzyme to Ca^2+^ release channels and myofilament regulatory proteins (Kirchhefer et al., 2014a). Although there is no change in PP2A activities in the ICM and DCM samples, the total protein phosphatase activities and PP1 activities increases with reduced phosphorylation on cTnI (Wijnker et al., 2011). Hyperphosphorylation of ryanodine by enhanced β-adrenergic stimulation and reduced phosphatase activities results in “Ca^2+^ leak” from sarcoplasmic reticulum in failing heart (Marx et al., 2000; Reiken et al., 2001).

### Reduced PP2A activities are associated with arrhythmia and atrial fibrillation (AF)

As reduced density of L-type Ca^2+^ current is characteristic of AF, increased PP2A activities were considered as an cause for the cardiac condition (Christ et al., 2004). Further analysis indicates that reduction of L-type calcium current density is due to a transcriptional downregulation of the pore forming alpha (1c)-subunit of LTCC, while single channel peak average current is 1.7-fold higher in AF than the control due to a 3.1-fold higher open probability of LCC. Inhibition of PP2A by okadaic acid only increases Ica in control but not in AF, suggesting phosphorylation of LCC in AF is high (Klein et al., 2003). Down regulation of PP2A-Bα by microRNA miR-1 is associated with elevated phosphorylation of RyR at CamKII site, but not the PKA sites with enhanced frequency of spontaneous Ca^2+^ sparks and arrhythmogenic oscillations of intracellular Ca^2+^(Terentyev et al., 2009).

### Post-translational modifications and mutation of PP2A associated with heart failure

Kirchhefer et al. reported that Bα of PP2A is phosphorylated at Ser41 by PKC α and phosphorylation at this site lead to reduction of the phosphatase activities. In failing human heart, phosphorylation of Bα is 7-fold higher (Kirchhefer et al., 2014b). The A subunit is also phosphorylated and phosphorylation attenuates assembly of PP2A heterotrimer and reduces PP2A activities characterized by increased phosphorylation occurred to a large number of proteins in cells expressing the peudophosphorylated constructs. Unlike phosphorylated Bα, in a rat model of heart failure phosphorylation at this subunit is reduced leading to higher PP2A activities. In transgenic mice expressing a truncated A subunit that is a dominant negative mutant disrupting normal PP2a assembly, dilated cardiomyopathy developed with increased end-diastolic and end-systolic dimensions and decreased fractional shortening (Brewis et al., 2000).

### The roles of PP2A in sensitizing β-adrenergic stimulation

Loss of response to β-adrenergic stimulation is a hall mark of end stage heart failure. Previously, it is believed that increased phosphatase activity is a major cause for desensitizing β-adrenergic stimulation as the β-adrenergic stimulation are effectively and rapidly damped by enhanced phosphatase activities. Accumulating evidence suggest that this may not be true because in failing heart, phosphorylation on L-type Ca^2+^ channels, ryanodine receptors and NCX are usually high. Phosphatases, especially PP2A can make them more responsive to β-adrenergic signals by bringing down phosphorylation levels. Recent studies by Zheng et al suggest that pyruvate restores β-adrenergic sensitivity of L-type Ca^2+^ channels in failing rat heart through PP2A (Zheng et al., 2013).

## Perspective

Structural diversity and complex regulation of PP2A constitute a significant challenge in understanding its function in the heart. Emerging evidence begins to point out connections between specific PP2A heterotrimers and their protein substrates in cardiomyocytes, but definitive results are still scarce. Application of general PP2A inhibitors for heart diseases may not be applicable as these inhibitors usually are tumorigenic. However, cardiac conditions including heart failure may become ameliorated by elevating PP2A activities. FTY720 (fingolimod), a FDA recently approved drug activates PP2A and target novel anti-adrenergic signaling pathways mediated by Pak1 (Egom et al., 2010). FTY720 protect heart against ischemia-reperfusion induced arrhythmia and has demonstrated anti-hypertrophic effect in mouse TAC model (Liu et al., 2011b, 2013). Its roles in modulation of Ca^2+^ transient in failing heart in animal models and in humans deserve further investigation.

### Conflict of interest statement

The authors declare that the research was conducted in the absence of any commercial or financial relationships that could be construed as a potential conflict of interest.
